# Solvent extraction of uranium from leach solutions obtained in processing of Polish low-grade ores

**DOI:** 10.1007/s10967-016-5029-5

**Published:** 2016-09-14

**Authors:** Katarzyna Kiegiel, Anna Abramowska, Paweł Biełuszka, Grażyna Zakrzewska-Kołtuniewicz, Stanisław Wołkowicz

**Affiliations:** 1 Institute of Nuclear Chemistry and Technology, Dorodna 16, 03-195 Warsaw, Poland; 2 Polish Geological Institute National Research Institute, Rakowiecka 4, 00-975 Warsaw, Poland

**Keywords:** Uranium, Sandstones, Geochemical analysis, Solvent extraction

## Abstract

Solvent extraction of uranium from acidic and alkaline post-leaching liquors that were obtained by leaching of Polish ores is reported in this paper. The stripping of uranium from organic to aqueous phase was also studied. The synergistic mixture of 2-diethylhexylphosphoric acid (D2EHPA) and tri-*n*-butylphosphate (0.2 M:0.2 M) was found as a good extracting agent for uranium. Recovery of uranium was reached even 98 %. The effect of such parameters like uranium concentration and concentration of reagents used in the experiments was evaluated in advance by using a model uranium solutions.

## Introduction

Uranium was extracted from the raw material in complex hydrometallurgical processes involving many separation steps. Processes such as solid–liquid extraction, solvent extraction, and ion exchange are applied to obtain pure triuranium octaoxide (U_3_O_8_) from uranium ore [1]. Since in most of uranium minerals uranium is accompanied by other heavy metals, post-leaching liquors usually contain a mixture of different metal ions that should be separated from UO_2_^2+^. Solvent extraction is a versatile technique for separating ionic solutes. The uranyl ion forms complexes with various organic chelatic agents. Nowadays, the literature reports a great variety of extractants that have been used for the extraction of uranium from aqueous solutions [2–4]. The most of them are the nitrogen-based, phosphorous-based and sulphur-based extractants. Among these neutral organophosphorus extractants tri-*n*-butylphosphate (TBP) probably received the most attention and use of this solvent on a commercial scale for the recovery of uranium (VI) from its ores and spent nuclear fuel is well known [5]. However, the selectivity of TBP is not high, as well as its radiolytic stability. Other organophosphorus extractants, including bis-2-ethylhexyl phosphoric acid (HDEHP) are applied in technology of uranium production. The aim of these studies was a selection of the extracting agents appropriate for the recovery of uranium from acidic and alkaline post-leaching liquors that were obtained by leaching of Polish ores. The raw extractants, like e.g.: tributylphosphate (TBP), di(2-ethylhexyl) phosphoric acid (D2EHPA), trioctylphosphine oxide (TOPO), triethylamine (TEA), tri-*n*-octylamine (TnOA), etc. were tested, and separation of uranium from other metals present in leach solutions, and efficiencies of their extraction were determined. The effect of type of extractant, sulphuric acid and uranium concentrations on the extraction process from model solutions was investigated. The results of these experiments were further used for the extraction of uranium from real post-leaching liquors. The use of different reagents as strip solutions selected on the basis of the literature data was also investigated [6, 7]. Stripping agents such as sodium carbonate solution, ammonium carbonate solution, sulphuric acids were tested for recovery of uranium from the organic phase.

One of the main elements in the development of nuclear energy is knowledge concerning potential sources to supply uranium for nuclear fuel production. In every country the problem of security if raw resources, also in energy aspect, is the subject of geological surveys. In parallel to geological examination usually the research on the technology of recovery of useful ingredients is carried on. The same approach is in Poland. Conducted geological research, throughout Poland, allow to state that there are no reach and easily accessible uranium resources. For that reason into the researches included low grade uranium resources located in hard geological conditions. This study is one of the part of research on a much broader scale which also included aspects of uranium mining profitability of this type of deposit in Poland, leading to obtain yellow cake.

The most prospective uranium mineralization on the Polish territory is the lower and middle Triassic rocks of the central parts of Peribaltic Syneclise. However, it is situated at depths of at least 750 m so they have to be treated as prognostic or perspective [8, 9]. The post-leaching solution examined in this study were obtained by leaching of these rocks.

## Geochemical analysis of uranium-bearing Triassic sandstones from Peribaltic Syneclise

The technological research based on natural rock samples have to be preceded by geochemical studies with determination of forms of useful element, its relation to other elements, both trace and major, and thus to examine the broader chemical context of environment in which examined element, in this case uranium, is present. Natural rock material is usually very diverse in geochemical aspect and the selection of one of known methods for processing have to be preceded by detailed geochemical tests.

Uranium mineralization in sandstones of Peribaltic Syneclise has typical epigenetic character. The characteristic features of sandstone-type uranium deposits are the significant vertical and horizontal variation zonation as well as zonal distribution of the trace elements associated with uranium.

From the point of view of uranium recovery technology its correlations with other metals, which may be the subject of simultaneous recovery during technological processes is very important. This might have an impact on improving the profitability of exploitation and processing of uranium ore because other metals which can be recovered will be co-product.

**Fig. 1:**
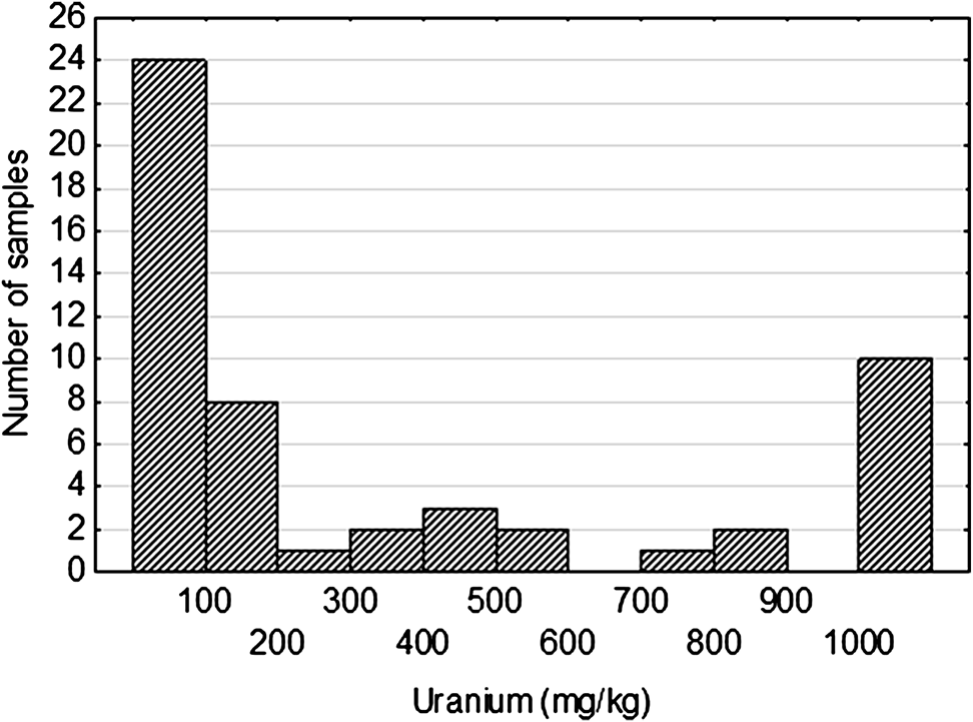
Uranium distribution in Triassic sandstones

The concentration of metals was determined by using inductively coupled plasma mass spectrometry (ICP-MS). Uranium content in the analyzed samples was highly variable and ranges from 4.2 ppm to nearly 1.5 %. The reason for this was that the samples come from strongly mineralized zones as well as from surrounding gangue rocks. The arithmetic mean of uranium content in the analyzed samples was 804 ppm, while the geometric mean was several times lower (138 ppm). The standard deviation characterized variability of set was very high and equals 2228 ppm. Histogram (Fig. 1) clearly illustrates polygenetic character of uranium mineralization.

**Fig. 2:**
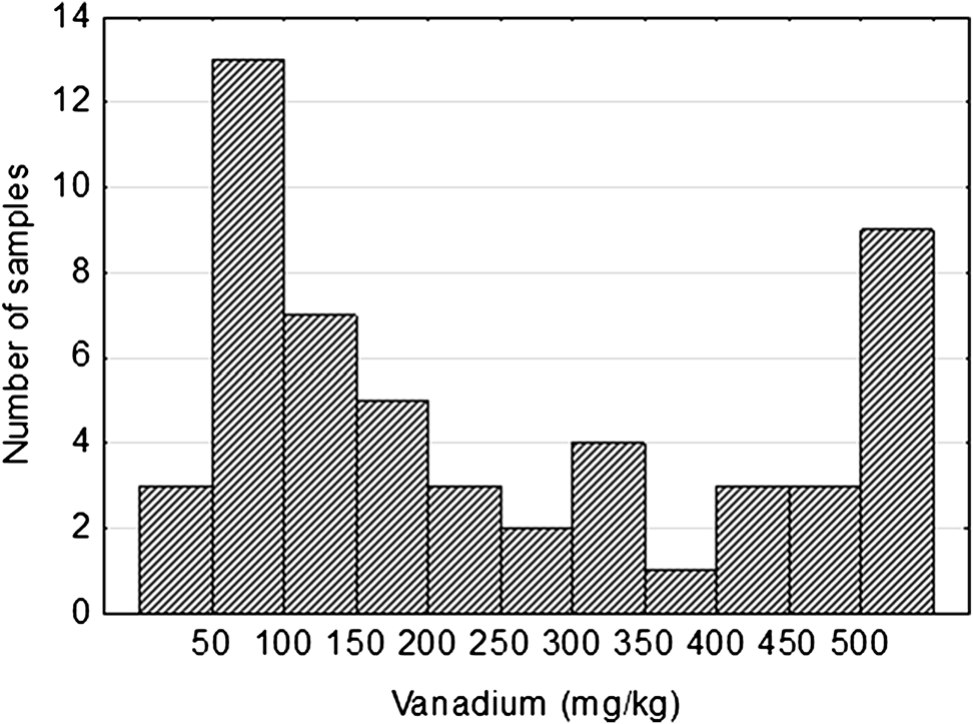
Vanadium distribution in Triassic sandstones

Vanadium is an element which often accompanied by the uranium in this type deposits. Its content in the studied population was also highly variable and ranges from 33 ppm to 0.46 %. The arithmetic mean was 362 ppm and the geometric mean—195 ppm. The histogram of vanadium distribution is multimodal distribution (Fig. 2). It indicated the multi-stage formation of vanadium concentration in the rock. Main modal value was located in the class 50–100 ppm, which substantially corresponded to the background value: about 35 ppm for sandstones and 130 ppm for claystones. There were still quite numerous group of samples characterized by elevating content of vanadium, exceeding 300 ppm. Comparison of U and V distribution showed their similarity.

**Fig. 3:**
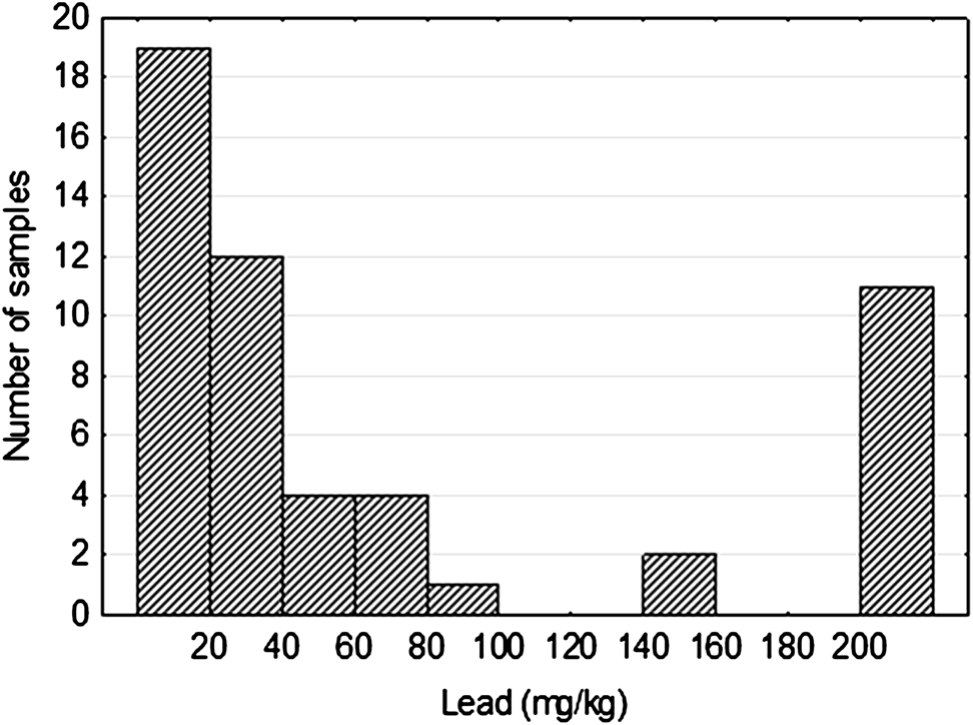
Lead distribution in Triassic sandstones

Selenium is an element associated with uranium mineralization in Triassic rocks of Peribaltic Syneclise. Its content ranges from <1 ppm (detection limit of the analytical method) to more than 0.43 %. The arithmetic average of Se content of this element was about 110 ppm, and the geometric mean is 4.5 ppm. Spot accumulation of selenium in the rock was manifested by the presence of its own mineral–clausthalite, which was identified in the mineralogical study. The lead content ranged from 6.8 ppm to 0.62 %. The arithmetic mean of Pb content was 323 ppm, and the geometric mean was several times lower and was equal to 49 ppm. Histogram of the lead distribution is similar to the uranium one (compare Figs. 1, 3). It is multimodal, with clearly defined population samples, covering approximately 25 % of the samples with relatively high contents of lead (200 ppm). This similarity might indicate the radiogenic origin of a large part of this element.

**Fig. 4:**
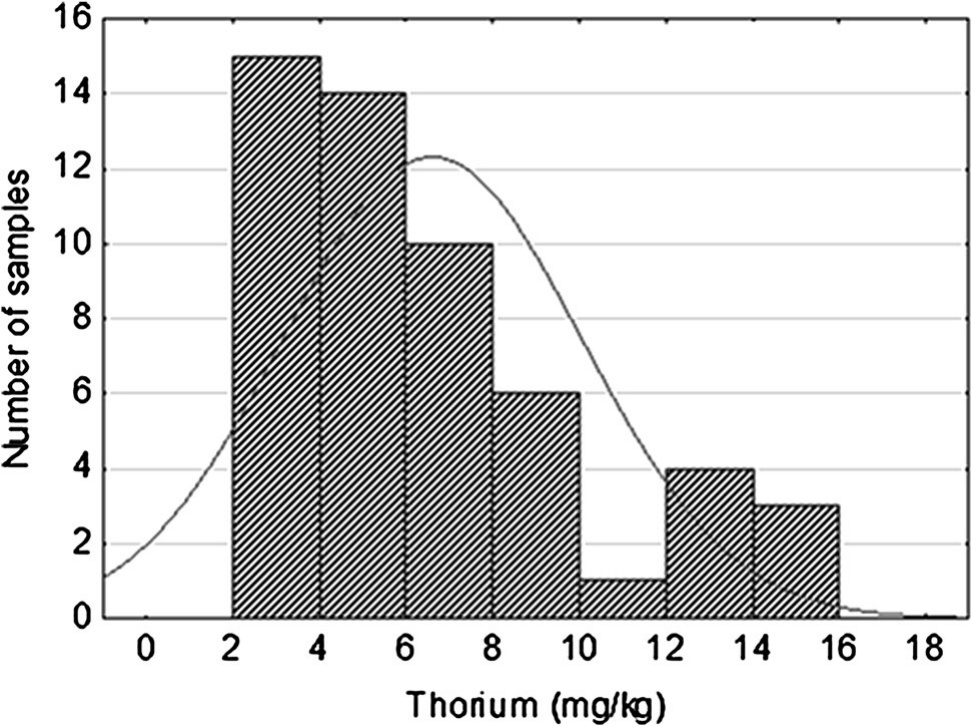
Thorium distribution in Triassic sandstones

Thorium content in examined samples was low, ranging from 2.5 to 15.2 ppm. The arithmetic mean of Th content was 6.6 and the geometric mean of 5.8 ppm. These contents are typical of sandstone and reflect syngenetic nature of thorium presence in studied rocks. Histogram of distribution is practically unimodal, skewed right, the modal value is located in the bin from 2 to 4 ppm (Fig. 4). Differentiation of thorium content corresponds to the lithological variability of studied rock formation consisting mainly of sandstones with finer-grained inserts and interbeddings. Typically Th/U ratio in sandstones varies from 3 to 4. Using this ratio syngenetic uranium concentration should vary between 1 and 5 ppm U. Thus, as a result of epigenetic processes mineralizing enrichment factor of uranium reached a few thousand.

**Fig. 5:**
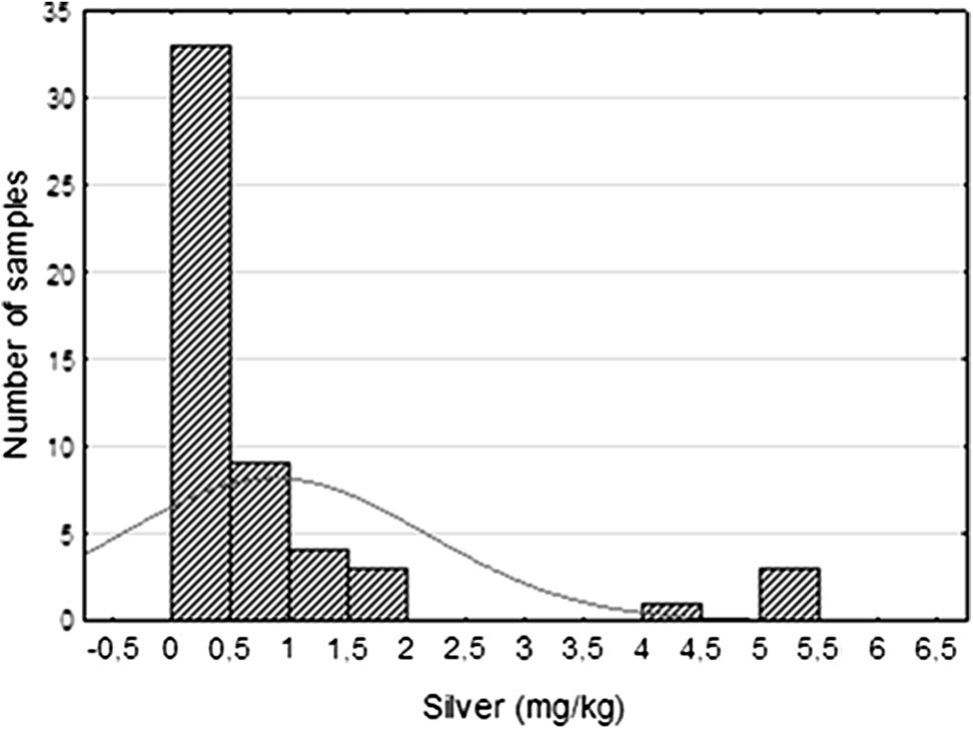
Silver distribution in Triassic sandstones

From the other elements worth noting the silver. Its content ranged from 0.1 to 5.5 ppm. The arithmetic mean of Ag contents was 0.86 ppm and the geometric mean was 0.47 ppm. Histogram of distribution indicates the presence of a dominant population with modal value located in the bin from 0 to 0.5 ppm, but more than 25 % of the samples comprise silver in an amount from 0.5 to 2 ppm (Fig. 5). Furthermore, there was another group of samples (8 % of population) with silver contents ranging from 4 to 5.5 ppm. This indicated the presence of rocks clearly epigenetically enriched in silver.

In the studied rocks uranium showed the strongest correlation with lead (0.92), yttrium (0.93), silver (0.76), copper (0.75), antimony (0.70), and cobalt (0.44). It was negatively correlated with barium (−0.43) and strontium (−0.36) (Table 1). There was no correlation with vanadium, despite the fact that both of these elements certainly had epigenetic origin. This was due to the fact that uranium and vanadium anomalies have a different geometry: vanadium occurs mainly claystones and siltstones while the highest concentrations of uranium are associated with poorly cemented sandstones. From the main components of rocks uranium showed a positive and significant correlation only with P_2_O_5_ (Table 2).

**Table 1:** Correlation coefficient of selected trace elements in U-bearing Triassic rocks of Peribaltic Syneclise (n = 53)

	V	Cr	Co	Ni	Cu	Zn	As	Se	Sr	Mo	Ag	Cd	Sn	Sb	Ba	Pb	U	Sc	Y	Nb	Ta	Th
V	1.00	0.78	−0.03	0.04	0.09	0.38	0.72	0.93	−0.31	−0.07	0.59	0.45	0.02	0.17	0.14	0.33	0.17	0.17	0.00	0.03	0.01	0.23
Cr	0.78	1.00	−0.02	0.02	0.04	0.25	0.71	0.88	−0.07	−0.04	0.45	0.28	−0.08	0.17	0.02	0.18	0.02	0.24	−0.04	−0.16	−0.14	0.10
Co	−0.03	−0.02	1.00	0.73	0.76	0.22	0.09	−0.08	−0.08	0.13	0.56	0.16	0.33	0.23	−0.43	0.31	0.44	0.46	0.43	0.27	0.29	0.21
Ni	0.04	0.02	0.73	1.00	0.73	0.75	0.16	−0.09	0.05	0.48	0.38	0.64	0.77	0.20	−0.26	0.20	0.20	0.76	0.29	0.70	0.70	0.78
Cu	0.09	0.04	0.76	0.73	1.00	0.55	0.24	0.00	−0.11	0.25	0.71	0.45	0.67	0.52	−0.49	0.71	0.75	0.59	0.77	0.53	0.49	0.45
Zn	0.38	0.25	0.22	0.75	0.55	1.00	0.44	0.21	0.00	0.44	0.37	0.84	0.85	0.35	−0.04	0.31	0.17	0.73	0.22	0.76	0.73	0.93
As	0.72	0.71	0.09	0.16	0.24	0.44	1.00	0.77	−0.21	0.11	0.64	0.33	0.16	0.67	−0.10	0.42	0.34	0.24	0.26	0.06	0.03	0.19
Se	0.93	0.88	−0.08	−0.09	0.00	0.21	0.77	1.00	−0.20	−0.04	0.57	0.33	−0.14	0.18	0.12	0.30	0.14	0.05	0.03	−0.18	−0.18	0.04
Sr	−0.31	−0.07	−0.08	0.05	−0.11	0.00	−0.21	−0.20	1.00	0.00	−0.33	0.02	0.04	−0.19	−0.40	−0.25	*−0.36*	0.16	−0.08	−0.23	−0.13	0.00
Mo	−0.07	−0.04	0.13	0.48	0.25	0.44	0.11	−0.04	0.00	1.00	0.08	0.61	0.52	0.02	0.00	0.03	0.03	0.35	0.23	0.52	0.40	0.57
Ag	0.59	0.45	0.56	0.38	0.71	0.37	0.64	0.57	−0.33	0.08	1.00	0.37	0.23	0.52	−0.36	0.72	0.76	0.39	0.65	0.16	0.15	0.19
Cd	0.45	0.28	0.16	0.64	0.45	0.84	0.33	0.33	0.02	0.61	0.37	1.00	0.74	0.11	−0.04	0.32	0.11	0.52	0.21	0.65	0.59	0.82
Sn	0.02	−0.08	0.33	0.77	0.67	0.85	0.16	−0.14	0.04	0.52	0.23	0.74	1.00	0.41	−0.15	0.44	0.31	0.58	0.43	0.86	0.80	0.81
Sb	0.17	0.17	0.23	0.20	0.52	0.35	0.67	0.18	−0.19	0.02	0.52	0.11	0.41	1.00	−0.34	0.72	0.70	0.18	0.67	0.21	0.13	0.07
Ba	0.14	0.02	−0.43	−0.26	−0.49	−0.04	−0.10	0.12	−0.40	0.00	−0.36	−0.04	−0.15	−0.34	1.00	−0.33	−0.43	−0.29	−0.56	0.10	0.05	0.08
Pb	0.33	0.18	0.31	0.20	0.71	0.31	0.42	0.30	−0.25	0.03	0.72	0.32	0.44	0.72	−0.33	1.00	0.92	0.15	0.87	0.23	0.12	0.07
U	0.17	0.02	0.44	0.20	0.75	0.17	0.34	0.14	−0.36	0.03	0.76	0.11	0.31	0.70	−0.43	0.92	1.00	0.18	0.93	0.17	0.08	−0.02
Sc	0.17	0.24	0.46	0.76	0.59	0.73	0.24	0.05	0.16	0.35	0.39	0.52	0.58	0.18	−0.29	0.15	0.18	1.00	0.23	0.50	0.49	0.74
Y	0.00	−0.04	0.43	0.29	0.77	0.22	0.26	0.03	−0.08	0.23	0.65	0.21	0.43	0.67	−0.56	0.87	0.93	0.23	1.00	0.20	0.11	0.05
Nb	0.03	−0.16	0.27	0.70	0.53	0.76	0.06	−0.18	−0.23	0.52	0.16	0.65	0.86	0.21	0.10	0.23	0.17	0.50	0.20	1.00	0.96	0.84
Ta	0.01	−0.14	0.29	0.70	0.49	0.73	0.03	−0.18	−0.13	0.40	0.15	0.59	0.80	0.13	0.05	0.12	0.08	0.49	0.11	0.96	1.00	0.81
Th	0.23	0.10	0.21	0.78	0.45	0.93	0.19	0.04	0.00	0.57	0.19	0.82	0.81	0.07	0.08	0.07	−0.02	0.74	0.05	0.84	0.81	1.00

Vanadium showed the strongest correlation with selenium (0.93), chromium (0.78), and arsenic (0.72) and slightly weaker with silver (0.59), cadmium (0.45) and zinc (0.38). From the main components of rock showed positive correlated with TiO_2_ (0.52), whereas showed significant negative correlations with CaO (−0.37). Selenium had a strong correlation with vanadium, chromium (0.88), arsenic (0.77) and silver (0.57). Lead had a very strong correlation with uranium (0.92) and yttrium (0.87), slightly lower correlations with silver and antimony (0.72), copper (0.71), tin (0.44), and arsenic (0.42). From the main components of rocks lead showed a positive and significant correlation with P_2_O_5_ and Na_2_O.

Thorium had a numerous and relatively high correlations ranging from 0.93 to 0.45, with such elements zinc, nickel, copper, molybdenum, cadmium, tin, scandium, yttrium, niobium and tantalum. The significant part this was due to the presence of dark accessory minerals in studied rocks. This was also confirmed by numerous positive thorium correlations with the main components of rocks, such as TiO_2_, Al_2_O_3_, K_2_O, MgO and Fe_2_O_3_, and a significant negative correlation with CaO, which component was largely in the form of carbonate, epigenetic cement (Table 2).

**Table 2:** Correlation coefficient of selected trace elements and main components in U-bearing Triassic rocks of Peribaltic Syneclise (n = 53)

	SiO_2_	TiO_2_	Al_2_O_3_	Fe_2_O_3_	MnO	MgO	CaO	Na_2_O	K_2_O	P_2_O_5_	(SO_3_)	(Cl)	(F)	LOI
V	0.33	0.52	0.19	−0.04	−0.31	−0.14	−0.37	0.25	0.23	0.23	−0.09	0.50	−0.29	−0.34
Se	0.21	0.35	0.00	−0.06	−0.15	−0.14	−0.20	0.13	0.05	0.20	−0.09	0.38	−0.19	−0.20
Ag	0.20	0.52	0.17	0.18	−0.19	0.16	−0.34	0.26	0.22	0.66	0.39	0.41	−0.47	−0.27
Pb	0.25	0.22	0.21	0.03	−0.30	−0.09	−0.35	0.37	0.29	0.40	−0.05	0.44	−0.34	−0.29
U	0.26	0.23	0.07	0.05	−0.25	−0.06	−0.33	0.33	0.20	0.55	0.16	0.40	−0.35	−0.30
Th	0.02	0.76	0.92	0.49	−0.33	0.47	−0.39	0.13	0.65	0.35	0.10	0.11	−0.55	−0.17
SiO_2_	1.00	0.38	0.23	−0.36	−0.90	−0.56	−0.91	0.87	0.66	0.12	−0.19	0.83	−0.32	−0.98
TiO_2_	0.38	1.00	0.67	0.32	−0.53	0.18	−0.63	0.39	0.63	0.70	0.25	0.41	−0.66	−0.51
Al_2_O_3_	0.23	0.67	1.00	0.44	−0.55	0.33	−0.59	0.42	0.86	0.25	−0.06	0.29	−0.60	−0.38
Fe_2_O_3_	−0.36	0.32	0.44	1.00	0.08	0.44	0.04	−0.17	0.16	0.26	0.28	−0.12	−0.50	0.19
MnO	−0.90	−0.53	−0.55	0.08	1.00	0.36	0.96	−0.85	−0.85	−0.17	0.19	−0.78	0.53	0.94
MgO	−0.56	0.18	0.33	0.44	0.36	1.00	0.26	−0.41	−0.01	0.21	0.47	−0.45	−0.26	0.45
CaO	−0.91	−0.63	−0.59	0.04	0.96	0.26	1.00	−0.89	−0.88	−0.29	0.07	−0.82	0.59	0.97
Na_2_O	0.87	0.39	0.42	−0.17	−0.85	−0.41	−0.89	1.00	0.78	0.20	−0.12	0.78	−0.45	−0.90
K_2_O	0.66	0.63	0.86	0.16	−0.85	−0.01	−0.88	0.78	1.00	0.23	−0.12	0.61	−0.60	−0.77
P_2_O_5_	0.12	0.70	0.25	0.26	−0.17	0.21	−0.29	0.20	0.23	1.00	0.37	0.11	−0.46	−0.22
(SO_3_)	−0.19	0.25	−0.06	0.28	0.19	0.47	0.07	−0.12	−0.12	0.37	1.00	−0.05	−0.26	0.11
(Cl)	0.83	0.41	0.29	−0.12	−0.78	−0.45	−0.82	0.78	0.61	0.11	−0.05	1.00	−0.36	−0.84
(F)	−0.32	−0.66	−0.60	−0.50	0.53	−0.26	0.59	−0.45	−0.60	−0.46	−0.26	−0.36	1.00	0.46
LOI	−0.98	−0.51	−0.38	0.19	0.94	0.45	0.97	−0.90	−0.77	−0.22	0.11	−0.84	0.46	1.00

## Experimental

### Reagents and solutions

The chemicals and reagents used in these studies were used as received. Uranyl nitrate of analytical reagent grade were supplied by Chemapol Praha. The extracting agents: tributylphosphate (TBP), di (2-ethylhexyl)phosphoric acid (D2EHPA), trioctylphosphine oxide (TOPO), triethylamine (TEA), tri-*n*-octylamine (TnOA) and kerosene were Aldrich products. All other reagents used were analytical or reagent grade.

A model solution of uranium was prepared by dissolving a fixed amount of UO_2_(NO_3_)_2_·6H_2_O in 5 % sulfuric acid.

### Uranium leach solution

The uranium liquors (sulphuric and carbonate) used in the experiments were obtained by leaching Polish uranium ores: Triassic sandstones using sulphuric acid or sodium carbonate and bicarbonate solution [10, 11]. The leaching liquors contained the following metals:

1. The solution obtained after sulphuric acid leaching: U:25.32 µg ml^−1^, Th:0.06 µg ml^−1^, Cu:0.53 µg ml^−1^, Co:15.00 µg ml^−1^, Mn:4.75 µg ml^−1^, Zn:3.88 µg ml^−1^, Cr:1.83 µg ml^−1^, La:0.20 µg ml^−1^, V:3.04 µg ml^−1^, Yb:0.02 µg ml^−1^, Ni:1.04 µg ml^−1^, Fe:71.18 µg ml^−1^.
2. The solution obtained after alkaline leaching: U:19.24 µg ml^−1^, Mn:0.13 µg ml^−1^, Zn:0.37 µg ml^−1^, V:0.58 µg ml^−1^.

### Extraction/stripping experiments

The extraction and stripping experiments were carried out in plastic (polypropylene) or glass tubes under mechanical agitation (500 rpm), at room temperature (25 ± 2 °C). Kinetic studies showed that extraction equilibrium was reached after ca. 15 min. However, in all extraction and stripping experiments a contact time of 30 min was chosen for ensuring that the equilibrium was reached. The organic:aqueous phase volume ratio variation was fixed at 1:1. The organic phase used as a solvent for extraction was composed of kerosene as diluent for extracting agents. The acidity of aqueous phase (post-leaching liquors) before extraction experiments was adjusted to pH that was indicated in Table 3 by using 2 M H_2_SO_4_. Following phase contact and reaching equilibrium, the aqueous and organic phases were separated by means of separation funnel and then analysed by inductively coupled plasma mass spectrometry (ICP-MS) [12].

**Table 3:** Effect of extractant concentration and pH of initial aqueous phase on uranium extraction and stripping efficiencies

Entry	Extraction			Stripping					
				0.5 M Na_2_CO_3_		0.5 M (NH_4_)_2_CO_3_		7 M H_2_SO_4_	
	[D2EHPA]:[TBP]	PH	%E	%S	%R	%S	%R	%S	%R
1	0.2 M:0.2 M	6	99	Third phase was forming				–	–
2	0.2 M:0.2 M	3	99	88	87	97	96	29	28
3	0.2 M:0.2 M	1	99	93	92	94	93	–	–
4	0.2 M:0.07 M	10		Emulsion					
5	0.2 M:0.07 M	6	99	Third phase was forming				–	–
6	0.2 M:0.07 M	1	99	Third phase was forming				34	33
7	0.1 M:0.1 M	6	83	64	55	82	71	47	41
8	0.1 M:0.1 M	1	99	99^a^	98	99^a^	98	65	64

^a^2 days were needed for separation aqueous and organic phases

The extraction efficiency (%*E*) was calculated by the formula (1):

1 $$\% E{ = 100 }D_{\text{c}} / ( { }D_{\text{c}} { + }V_{\text{aq}} /V_{\text{org}} )$$

where *D*_c_ is the distribution ratio, defined as the ratio of concentration of metal in organic phase over its concentration in aqueous phase, *V*_aq_—aqueous phase volume, *V*_org_—organic phase volume [2]:

The stripping percentage, %*S* was determined by the relationship (2):

2 $$\% S = 100 D_{\text{s}} / (D_{\text{s}} { + }V_{\text{aq}} /V_{\text{org}} )$$

where *D*_s_ is the distribution ratio of metal in stripping phase over its concentration in organic phase [2].

%*R* percent of recovery of uranium in extraction/stripping process was determined by the relationship (3):

3 $$\% R = \left[ {\text{Metal in the stripping phase}} \right] \, / \, \left[ {{\text{Metal in post}} - {\text{leaching liquor}}} \right]\cdot 100\%$$

The all experiments were repeated several times in order to confirm the correctness of the obtained results. The relative errors were no more than 5 %.

### Analysis

The concentration of selected ions in the aqueous phase was determined by inductively coupled plasma mass spectrometry (ICP-MS) after diluting with H_2_O and HNO_3_ to the concentration suitable for ICP-measurement. The ICP-MS instrument ELAN DRC II (Perkin Elmer) with cross-flow nebulizer with Scott double-pass spray chamber and Ni cones was used.

During experiments pH was monitored using, IoLine pH combination electrode (type IL-MICRO-pHT-A-BNC-N) coupled with the Schott multiparameter measuring instrument ProLab 4000.

## Results and discussion

**Fig. 6:**
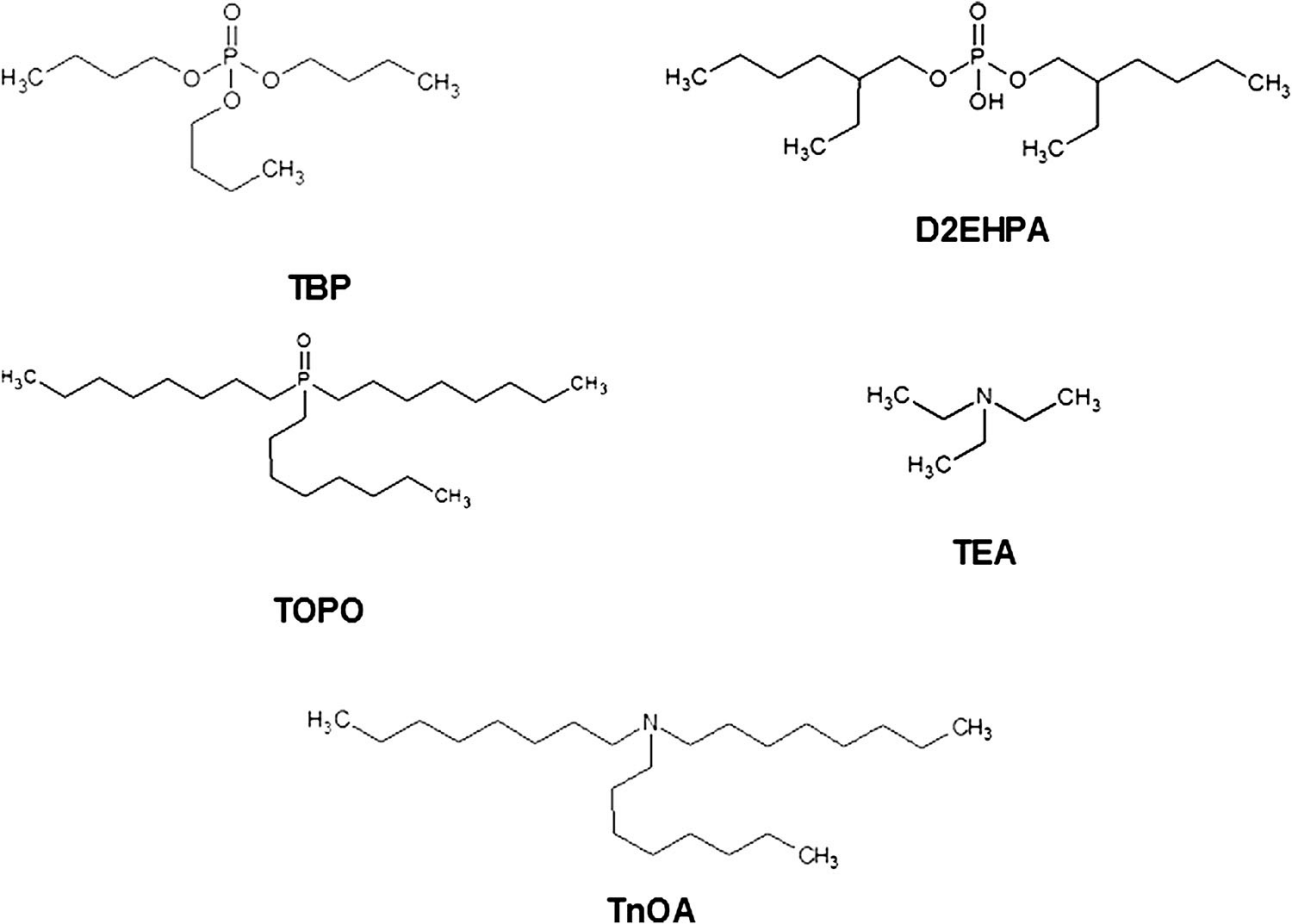
The extracting agents tested for the separation of uranium from the solution

The aim of preliminary studies was a selection of the extracting agents and extraction conditions appropriate for the recovery of uranium from post-leaching liquors. The effect of the type of extractant, sulphuric acid and uranium concentrations on the extraction process from model solutions containing uranium was investigated. The extracting agents, like e.g.: tributylphosphate (TBP), di (2-ethylhexyl) phosphoric acid (D2EHPA), trioctylphosphine oxide (TOPO), triethylamine (TEA) and tri-*n*-octylamine (TnOA) (Fig. 6) were tested with the model uranium solutions. Then, the recovery of uranium from post-leaching liquors by solvent extraction followed by stripping to aqueous phase was examined. The mixture of D2EHPA and TBP was found as a good extractant for uranium and the studies of extraction of uranium from ore-leaching liquors (sulphuric and carbonate) were carried out. The use a different reagents as strip solutions for uranium in organic phase was also investigated.

### Effect of time

**Fig. 7:**
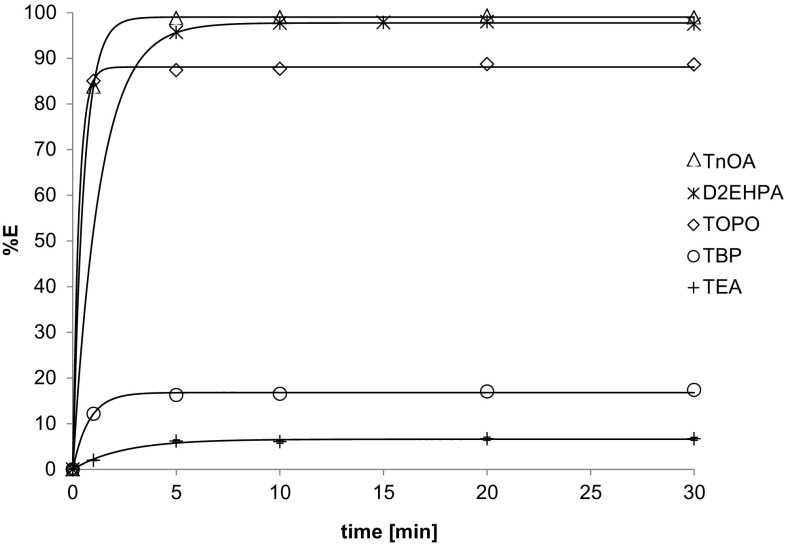
Time dependence of extraction efficiency of uranium for different extractants: 0.2 M (D2EHPA, TOPO, TEA, TBP, TnOA) in kerosene. The feed solution: 0.2 g U l^−1^ in 5 % H_2_SO_4_, *T* = 25 °C, *V*_aq_/*V*_org_ = 1

In order to determine the extraction kinetics for different extractants, the time dependence of the extraction efficiency of uranium was investigated and the results are plotted in Fig. 7. The extraction equilibrium was reached within 15 min for all the extractants tested.

### Effect of type of extractant

The tested organic solvents extracted uranium with different efficiency; according to %*E* they can be arranged in the following order (Fig. 7):

TnOA > D2EHPA > TOPO > TBP > TEA

Base on the above findings, two extractants: TnOA and D2EHPA were selected for extraction of uranium from post-leaching liquor. They were examined with the purpose of further stripping experiments. The results showed that uranium was extracted from the solution obtained after sulphuric acid leaching with high efficiency as expected (99 % for D2EHPA and 98 % for TnOA). The yield of extraction of some metals other than uranium was also high (Th: 99 %, Yb: 99 % for D2EHPA and Th: 51 %, V: 68 % for TnOA).

### Effect of sulphuric acid, uranium and extractant concentrations

**Fig. 8:**
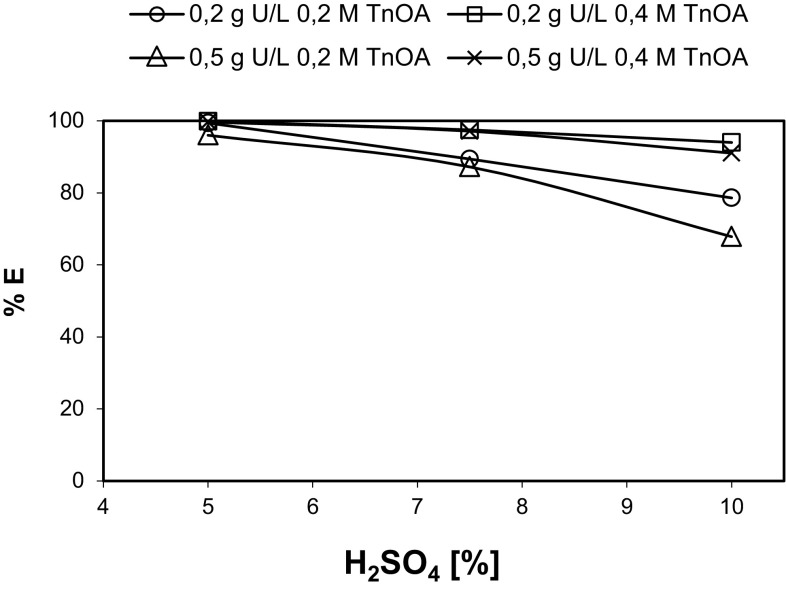
Effect of H_2_SO_4_ concentration on the extraction of uranium by TnOA in kerosene from H_2_SO_4_, *T* = 25 °C, *V*_aq_/*V*_org_ = 1

Figure 8 presents the extraction efficiency of uranium at different concentration of sulphuric acid. The experiments were performed for two initial concentrations of uranium: 0.2 and 0.5 g l^−1^ and two molar concentrations of TnOA: 0.2 M and 0.4 M. The results showed that the distribution ratios increase with the increase of extractants concentrations while they decrease with the increase of sulphuric acid concentration. The observed effect of decreased distribution ratios with increased acid concentration and increased distribution ratios with increased extractants (like e.g.: TBP, D2EHP) concentration was reported previously [13].

### Effect of aqueous/organic phase volume ratio

**Fig. 9:**
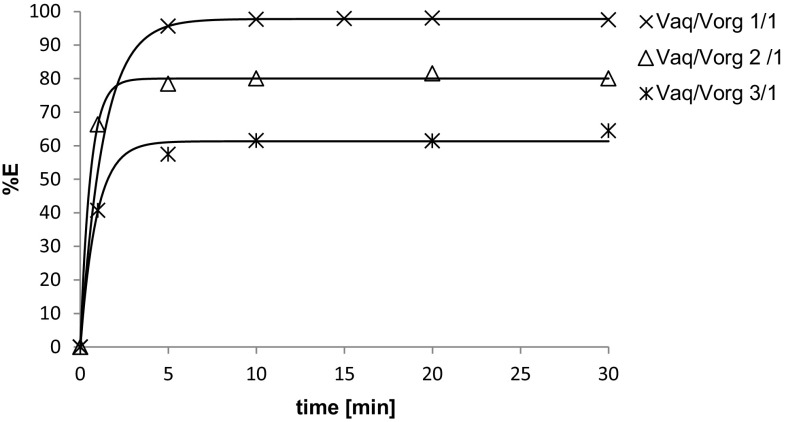
Effect of aqueous to organic phase volume ratio on the extraction of uranium by 0.2 M TnOA in kerosene from 5 % H_2_SO_4_; *T* = 25 °C, *V*_aq_/*V*_org_ volume ratio between aqueous phase and organic phase

Another process variable investigated was the aqueous/organic phase volume ratio. This study was carried out with 0.2 M TnOA as an extracting agent. The results showed that the efficiencies of extraction decreases with increasing aqueous/organic phase volume ratio (Fig. 9). Thus ratio of 1:1 was used for further studies of liquid–liquid extraction of uranium.

### Stripping of uranium

Once the metal ions have been extracted by the organic phase, they should be stripped back by an aqueous phase. The experiments revealed that the stripping of uranium from organic phase containing D2EHPA with sodium carbonate was not possible because of the separation of NaD2EHP in a third phase. On the other hand, the efficiency of the stripping from organic phase containing TnOA was not satisfactory; it was only 5–11 %. The further research showed that it was possible to avoid the third phase formation when the extractions were carried out with a synergistic mixture D2EHPA and TBP. The obtained results were very promising; the stripping was very efficient and almost complete back extraction of uranium was observed as it was shown below in Table 3.

### Effect of synergistic reagent and pH on extraction and stripping of uranium from the pregnant leach solutions

In order to choice of the extractant composition to be used in the extraction study of pregnant leach solutions, the solution of extractant were prepared in the following concentration: [D2EHPA]:[TBP] 0.2 M:0.2 M, 0.2 M:0.07 M, 0.1 M:0.1 M. The extraction percentages for different concentration of extractants are shown in Table 3. It was found that the optimal [D2EHPA]:[TBP] ratio is 0.2 M:0.2 M at pH 1 (entry 3). The higher pH is not recommended because the efficiency of stripping process is lower (entry 2). Using of lower concentration of reagents resulted in more difficult separation of phases during stripping experiment. The separation required long time (2 days) (entry 8) or was even impossible (entry 5 and 6). It worth to note that the summarized yield of extraction and stripping experiments (%*R*) reached even 98 % (entry 8). The use of sodium and ammonium carbonates as a stripping reagents is more preferable than using sulphuric acid.

**Fig. 10:**
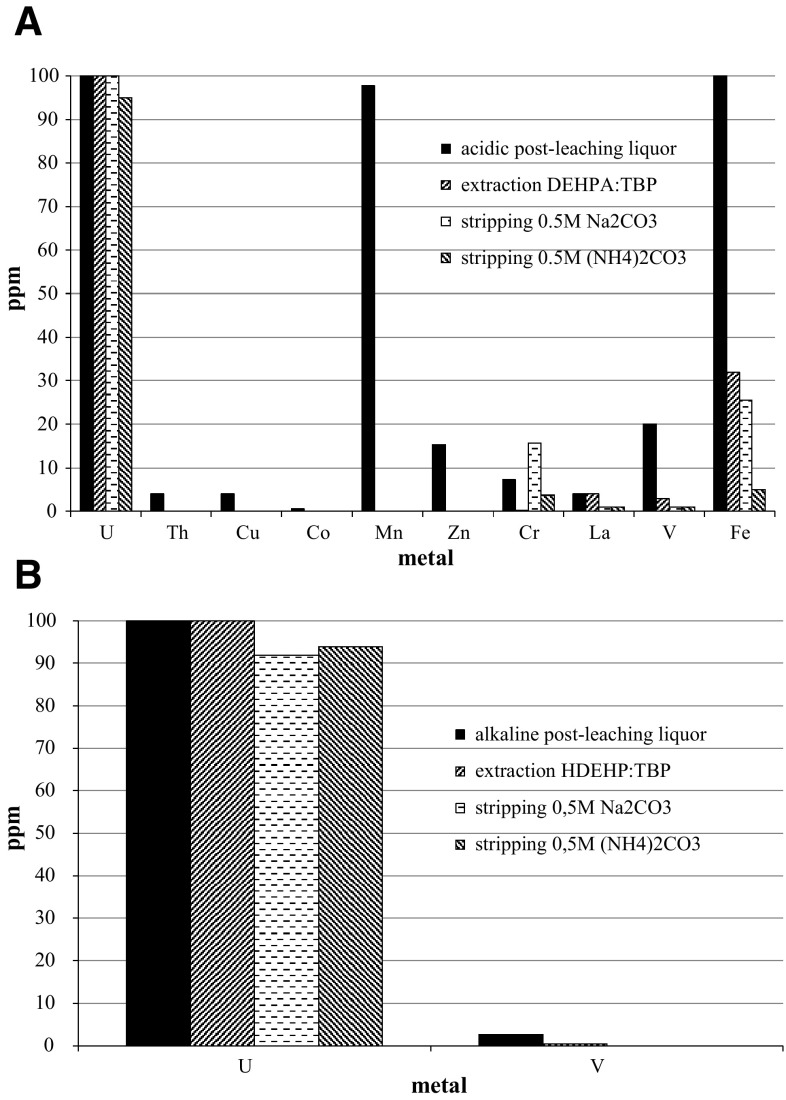
Extraction and stripping efficiencies of metals from: **a** acidic post-leaching liquor and **b** alkaline post-leaching liquor. Conditions: kerosene, *V*_aq_/*V*_org_ 1:1, [D2EHPA]:[TBP] 0.2 M:0.2 M, *T* = 25 °C

The extraction/stripping process of alkaline and acidic post-leaching liquors is illustrated in the Fig. 10. For this process apart from uranium, the extraction/stripping of the other elements were also examined. It worth to be noticed the purification of uranium from alkaline post-leaching liquor was almost completely. After the extraction step, only trace amounts of vanadium were present in organic phase. The stripping step gave pure uranium solution (Fig. 10a). The separation of uranium from accompanying metals from acid leaching solution was only partial (Fig. 10b). The highest extraction, stripping and recovery were obtained for uranium but in solution small amounts of other metals as lanthanum, vanadium and iron were detected.

Currently, not much solvent extraction methods have been reported for uranium recovery from alkaline post-leaching solutions. A number of quaternary amines were examined for recovery of uranium from carbonate leach solution but results were not satisfying. The common problem was the third phase formation. The solution of this problem was the extraction with an organic system comprising Aliquat 336 and isodecanol in Shellsol D70 [14]. The presented extraction of the pre-acidified alkaline post-leaching solution using synergestic mixture HDEHP-TBP mixture might be an alternative to the previously described system.

Further research may give more information about the mechanism of uranium extraction from post-leaching liquors by using organic extracting agents. Structural studies by such methods like XAFS [15–20] could give more information about the coordination environment of uranium reaction with the extractant.

## Conclusions

The synergestic extractant D2EHPA-TBP in kerosene as a diluent was used for recovery of uranium from the solutions obtained after leaching Polish uranium ores. The addition of TBP into the organic phase was found to be essential for preventing the formation of a third phase during the alkali treatment of a solvent containing D2EHPA. An organic phase composed of 0.2 M D2EHPA and 0.2 M TBP with kerosene as diluent is optimal for extraction of uranium at pH 1 and room temperature. The stripping of organic phase is very efficient with 0.5 M ammonium (or sodium) carbonate solution. The recovery of uranium reached even 98 %. High-purity uranium is recovered from the alkaline post-leaching liquor. However the single, one-stage extraction of uranium from acidic post-leaching liquors is not sufficient to separate pure uranium. The solvent extraction is a part of the research on the possibility of uranium extraction from domestic resources in Poland. It will be followed by ammonium or sodium diuranate precipitation, the precursors of yellow cake-U_3_O_8_.
